# Enhancing cancer prevention behaviors through social media: the role of source credibility and message characteristics in Ghana

**DOI:** 10.1186/s12889-025-23693-1

**Published:** 2025-07-16

**Authors:** Barikisu Issaka, Precious Allor, Ebenezer Ato Kwamena Aidoo, Sandra Freda Wood, Anne Amegbeha Amissah, Nancy Muturi

**Affiliations:** 1 Department of Advertising and Public Relations, Michigan State University, East Lansing, U.S.; 2 Department of Economics, Kansas State University, Manhattan, U.S.; 3 Communication Studies Department, University of Iowa, Iowa City, U.S.; 4 Department of Human Communication, Arizona State University, Tempe, U.S.; 5 Department of Communication and Information, University of Kentucky, Lexington, U.S.; 6 A.Q Miller School of Media and Communication, Manhattan, KS USA

**Keywords:** Cancer, Preventive behavior, Social media, Source credibility, Health belief model, Information processing theory, Message characteristics, Ghana

## Abstract

**Background:**

This study examines the influence of source and message characteristics on cancer prevention behavior in Ghana within the context of a social media platform. Drawing from the Information Processing Theory (IPT) and Health Belief Model (HBM), the research investigates the relationships between source credibility, message characteristics, social media usage, and key indices related to cancer prevention behavior.

**Methods:**

In this cross-sectional study, we surveyed 401 respondents across two social media platforms, namely Facebook and WhatsApp, which are the primary platforms utilized by the Zurak Cancer Foundation. We also chose to focus on these platforms because they reflect the dominant patterns of social media use in Ghana. Utilizing variables from the HBM and IPT, we conducted correlation and multiple regression analyses to examine how message, source, and channel characteristics influence cancer prevention behaviors. Additionally, we determined how IPT constructs, including perceived susceptibility, perceived severity, and self-efficacy, shape individual perceptions. Furthermore, we investigated the impact of both theories on cancer prevention behavior.

**Results:**

The results demonstrate associations between constructs from the Health Belief Model and Information Processing Theory and outcomes related to cancer prevention behavior. Notable associations were observed between social media usage and perceptions of source credibility, highlighting the role of digital engagement in shaping source evaluations. Additionally, message characteristics were linked to perceived message influence and behavioral outcomes, underscoring the importance of how health messages are framed and presented. The study also identified associations between the perceived usefulness of social media as a health information source and indicators of message influence and behavioral change.

**Conclusions:**

This research offers valuable insights for both scholars and practitioners involved in health communication and intervention strategies. It underscores the significance of selecting credible, attractive, and knowledgeable sources in the domain of cancer prevention for effective health campaigns. By tailoring messages to align with these source characteristics, practitioners can enhance the reach and effectiveness of their health communication efforts. Additionally, media organizations and content creators can leverage source credibility to amplify their social media presence, thereby attracting a broader audience and maximizing the impact of health-related content dissemination in an era dominated by social media.

**Supplementary Information:**

The online version contains supplementary material available at 10.1186/s12889-025-23693-1.

## Introduction

Cancer remains a significant global healthcare challenge, garnering consistent attention from healthcare professionals and researchers. In 2018, the world witnessed a substantial burden of cancer with 9.6 million cancer-related deaths and 18.1 million new cancer cases. Africa, including Ghana, contributed notably to these figures, with over 1 million new cases and almost 500,000 cancer-related deaths [1]. This highlights the urgent need for comprehensive cancer information, especially in regions like Ghana, where cancer registries are limited.

Ghana faces the additional challenge of inadequate and unreliable cancer data, primarily relying on records from medical institutions. Despite the limitations of these records, they reveal a concerning trend: cancer is rapidly becoming a leading cause of illness and death in the country [2, 3]. Medical reports consistently rank cancer among the top ten causes of death in Ghana [4]. Surprisingly, despite the growing burden of cancer and other non-communicable diseases in sub-Saharan Africa and specifically in Ghana, communicable diseases including HIV/AIDS, Ebola, Tuberculosis, and Malaria, continue to take precedence in healthcare priorities [5].

In Sub-Saharan Africa, a key contributor to high cancer rates and mortality is the lack of awareness and education about cancer [6]. Ghana has demonstrated that cancer education and awareness can significantly contribute to early detection efforts [7]. Over time, research has shown that communication plays a pivotal role in disseminating preventive health messages and driving behavior change [8, 9]. Campaigns using this approach have yielded positive outcomes by rectifying detrimental health behaviors and attitudes [10, 11]. Nonetheless, in Ghana, the majority of research has concentrated mainly on conventional media outlets like print, television, and radio [12, 13], while giving minimal attention to alternative sources of information, including social media. This is despite the widespread adoption of technology by health organizations for cancer communication and information dissemination across various platforms [14].

In an era characterized by rapid shifts in communication methods and the surge in online health information-seeking behaviors facilitated by participative internet use and social media, it is essential to comprehend these technologies and their impact on health communication [15]. Healthcare professionals increasingly recognize the significance of social media as a communication channel for researchers and health organizations to engage and educate their target audiences [16].

The untapped potential of social media, a powerful tool for health promotion globally.

 [17], presents a notable gap in Ghana’s cancer communication landscape. While the increasing use of social media by health organizations has transformed global health promotion strategies [18, 19], there is a significant lack of empirical research examining how social media is used for cancer prevention and education in the Ghanaian context. Despite the rise in internet and social media penetration in Ghana and the growing burden of cancer, most existing studies have focused on traditional media (e.g., radio, television, print), neglecting the evolving role of digital platforms in health messaging.

Furthermore, although international studies have shown that social media platforms can effectively enhance awareness, encourage preventive behaviors, and reduce stigma around cancer, little is known about how these platforms influence public perceptions, behavior change, or message processing in low-resource contexts like Ghana. This gap is particularly relevant given the high rates of late-stage cancer diagnoses and limited treatment access in the region. This study aims to fill this gap by exploring how social media content, particularly from Zurak Cancer Foundation (ZCF), a Ghanaian cancer advocacy organization, shapes individuals’ perceptions of cancer, influences preventive behavior, and challenges stigmatizing beliefs.

Based on the health belief model, the study examines perceptions of the severity and seriousness of the disease, their level of vulnerability, self-efficacy, and other motivational factors for adopting the recommended preventive behavior. In assessing communication efficiency, the study examines the dynamics of both source and message characteristics, including credibility and content through the lens of McGuire’s Information processing Model. Such understanding is crucial in guiding the design and implementation of evidence-based cancer prevention campaigns.

Amidst Ghana’s high internet and social media usage, we investigate how both the source and message characteristics within ZCF shape behavior and message impact. Additionally, we explore the roles of perceived susceptibility, self-efficacy, and perceived severity in message perception and platform efficacy. This research underscores the urgency and importance of studying cancer communication and individual perceptions as factors promoting health actions.

### Social media and health promotion

As a subset of the broader communications movement categorized as Web 2.0, social media refers to any type of networked information and communication technology (ICT) tool or platform that relies heavily on user participation [20, 21]. According to [22], “the medium is the message” (p.7), which means that every medium has unique features that profoundly influence how people relate to one another. Social media has emerged as a potential tool for various health-related initiatives, including health promotion [23].

Health promotion and social media have been described as a “beautiful marriage” due to how they are interdependent on each other [14]. In recent times, the use of social media for health promotion and public health education has been increasing due to the added advantages of accessibility and interactivity that come with the use of social media [14]. What sets social media apart from more traditional media like television, print, radio, and early websites is the audience’s capacity to provide feedback, share their own perspectives, and remix materials created by others [24]. Social media does not only provide two-way communication but connects people with similar health problems to help each other while enhancing access to health programs and services [25–27].

Research over the years has testified to the significant influence of social media in changing the behavior of individuals towards living a healthy lifestyle [28, 29]. Social media users, such as Twitter and Facebook users, have been found to acquire information on issues that are important to them through networking by intentionally subscribing, retweeting, or commenting [30]. Evidently, engaging in these activities online provides additional resources to people, however, to reduce possible misinformation, health institutions and other stakeholders or organizations have resorted to using the popularity of these social media platforms or outlets to promote health related information (diabetes, cancer, HIV/AIDS) to targeted audiences for behavioral change [31], awareness, as well as education [32, 33].

Interestingly, some of these organizations, institutions, or facilities are oriented toward more general health issues, whereas others are more specific to particular health conditions [34]. in their study, concluded that social media platforms are essential for creating health awareness because people are becoming knowledgeable about the causes, symptoms, prevention, and treatments of diseases. A systematic review conducted by [35] about young adults’ social media use for nutritional outcomes confirmed the significant role of social media in facilitating healthier eating choices and cooking recipe tips. Other studies on obesity [36], alcohol consumption [36], and smoking [37, 38] have demonstrated the influence of social networks on certain risk behaviors. Similarly [39], attributed the modification of health lifestyles among their participants in Nigeria to the influence of social media.

Social media platforms can help promote the early detection of illnesses including cancer through educational campaigns that raise awareness about the importance of screening and seeking timely medical attention [40]. Cancer health promotion studies show that social media platforms can disseminate credible and reliable information to patients, their families, and the wider public, thereby contributing to improved health outcomes [41–43]. Therefore, it is essential to consider the nuances of social media and cancer health promotion.

### Social media and cancer communication

Public perceptions significantly affect cancer treatment behaviors, necessitating efforts to combat negative perceptions and stigma for improved cancer care [44, 45]. Social media plays a vital role in shaping perceptions and challenging stigmatization. Negative attitudes towards cancer patients contribute to stigma and distrust in treatment effectiveness [46]. Social media is a potent tool for promoting cancer health, offering easy access to credible information and connection with healthcare professionals [47, 48]. Online health-seeking behavior, particularly among cancer patients and families, is rising, facilitated by platforms like Twitter, Facebook, and blogs [48, 49]. Active engagement on social media allows professionals to establish credibility as medical information sources [50–52]. Providing accurate and reliable content helps attract followers, strengthen patient-physician relationships, and boost confidence and engagement among patients [53]. Responsible use of social media by healthcare workers can enhance online health information, patient-physician relationships, and the overall quality of cancer care.

In a study conducted by [54], which assessed behavior related to cervical cancer using the Health Belief Model, it was discovered that social media played a more significant role in raising awareness compared to face-to-face interactions. This increased awareness had a significant impact on the perceived benefits of pap smear screenings and, consequently, influenced the behavior of approximately 73% of the women in the study, encouraging them to undergo pap smear screenings. In a similar vein [24], found that utilizing social media to acquire health-related information had a beneficial impact on individuals’ perceptions of the threat posed by colorectal cancer screening (CRC). Furthermore, it fostered positive expectations regarding CRC screening, and these perceptions subsequently influenced the uptake of the stool blood screening test. Evidently, social media provides a potent avenue to disseminate accurate cancer information, challenge misconceptions, and debunk stigmatizing beliefs. Despite extensive awareness campaigns, cancer remains a burden on Ghana’s healthcare system due to late detection and limited treatment access [55].

Social media can aid in promoting early detection through educational campaigns, raising awareness about screening importance, and encouraging timely medical attention [40]. Additionally, it facilitates the dissemination of information about available cancer treatment options, clinical trials, and supportive care services, improving access to comprehensive cancer care. Engaging social media users with informative and empathetic content, fostering supportive online communities, and encouraging dialogue to challenge stigmatizing beliefs can mitigate stigma, promote early detection, and improve access to quality cancer treatment in Ghana [40]. Several Ghanaian organizations, including Reach for Recovery, Cancer Connect Ghana, Breast Care International, Ghana Parents Association for Childhood Cancer, Lifeline for Childhood Cancer, Mmofra Foundation, Indian Women Association Accra, and Zurak Foundation, provide online resources to support cancer patients.

### Theoretical framework

Several theories inform health communication interventions that focus on cancer awareness, perceptions about the disease, and motivations for behavior change. This study draws from the IPT and HBM with the goal of understanding not only the perceptions people have about the disease but the most appropriate communication strategies to enhance awareness and motivate adoption of recommended behaviors.

### Information processing theory

The Information Processing Theory (IPT) as introduced by McGuire in 1989 provides valuable insights into how communication messages can effectively persuade individuals and drive behavior change. IPT focuses on five input factors from the sender’s perspective: source, message, channel, receiver, destination, and output factors from the receiver’s perspective, offering a comprehensive understanding of the communication process [56].

The source, whether an individual or organization, plays a pivotal role in communication effectiveness. Source characteristics include credibility, attractiveness, and power. Credibility hinges on expertise and perceived trustworthiness, while attractiveness comprises factors like liking, similarity, and familiarity [56, 57]. Previous research underscores the influence of source characteristics on message believability, behavior change, and persuasion [58, 59]. Our study examines the characteristics of ZCF and its influence on cancer prevention. Those characteristics include source credibility, attractiveness, trustworthiness, and expertise. Given that ZCF uses social media to disseminate information it is also crucial to understand the influence of social media usage on people’s perception of the source, which includes their familiarity and the extent to which they perceive them as a credible source.

Message characteristics including style, appeal type, argument structure, appearance, content, repetitiveness, and quantity are crucial components of IPT [56]. These attributes can significantly impact behavior change and message effectiveness. For instance, fear and guilt appeals, when effectively employed, motivate risk reduction [60]. Message appeals like hope appeals and varied formats, such as visual, voice, text-based, or infographics, can be more effective in shaping attitudes toward health behaviors [61, 62] [63]. highlights that fear inducing messages trigger two assessments: [1] an evaluation of the threat and [2] an assessment of the effectiveness of the suggested course of action. These evaluations lead to one of three reactions: no response, agreement, or dismissal. Our study aims to identify participants’ preferred message styles in ZCF’s cancer messages and their impact on cancer prevention behaviors.

Channels, mediums for message transmission (e.g., television, radio, newspapers, social media), are fundamental in IPT [56]. The choice of channel can significantly affect audience receptiveness to messages, with preferred channels playing a crucial role in engagement [64, 65]. In this study, we focus on social media, specifically Facebook as the channel. We investigate how social media, as a channel, influences receptiveness to cancer messages.

Receiver variables encompass the demographics of the intended audience, including personality, lifestyle, literacy, and audience participation. Understanding these variables is essential because individuals with varying personalities, attitudes, abilities, demographics, or ethnicities may react differently to persuasive message [66, 67] [68]. suggest that a successful health campaign aimed at the general public should be capable of influencing diverse individuals by tailoring the campaign to different high-risk subgroups with varying susceptibilities to different models of influence.

### Health belief model (HBM)

Our study integrates the Health Belief Model (HBM) to explore how communication channels, particularly social media, influence health message perceptions and receptiveness. The Health Belief Model (HBM), developed initially by social psychologists in the U.S. Public Health Service to explain people’s reluctance to participate in disease prevention and detection programs, centers around key concepts predicting individuals’ actions regarding health messages [69, 70]. These concepts include susceptibility, seriousness, benefits, barriers, and self-efficacy [71]. According to HBM, individuals must believe they are at risk, recognize the seriousness of the disease, perceive benefits in preventive actions, and assess barriers. Additionally, individual motivation plays a pivotal role in behavior change [69, 70].

In recent years, researchers have extended the HBM framework to analyze health content on social media platforms. For instance [72], used HBM-guided deep learning analysis to examine breast cancer screening beliefs on Twitter, finding that public discourse reflected concerns about susceptibility and benefits, thus validating HBM’s relevance in the digital space. Similarly [73], conducted a systematic review and meta-analysis showing that social media and mHealth interventions grounded in behavioral theories such as HBM improved participation in cancer screening programs, particularly when messages emphasized perceived benefits and addressed barriers. These findings underscore the importance of considering HBM constructs when assessing health message effectiveness in online settings.

Previous studies have used HBM constructs to understand their impact on health message perceptions [74]. found that low perceived susceptibility influenced Ghanaian women’s lack of engagement in breast cancer preventive measures, emphasizing the need for awareness campaigns [75]. demonstrated that message framing, whether loss-framed or gain-framed, influenced perceived susceptibility [76]. conducted a study on Hepatitis B virus perceptions among pregnant women in Uganda, revealing that high perceived self-efficacy correlated with behavioral intentions aimed at prevention. In our study, we incorporate HBM to examine its influence on message and source perception.

Based on the Health Belief Model, we aim to identify how IPT constructs determine individual perceptions, such as perceived susceptibility, perceived severity, and self-efficacy. We also aim to investigate how both theories influence cancer prevention behavior. Hence, we formulate the following hypotheses.

- H1: Social media usage is positively correlated with the perceptions of source credibility, attractiveness, source trustworthiness and source expertise.
- H2: Source credibility, attractiveness, trustworthiness, and source expertise were positively correlated with cancer prevention behavior.
- H3: Perception of the message is positively correlated with the influence of cancer prevention behavior.
- H4: Individuals who perceive social media as a reliable source of cancer information are more likely to be influenced by messages on this platform. Hence social media usage is expected to be positively correlated with the message influence.
- H5: Perception of the message is positively correlated with measures of the HBM model (i.e., Self-efficacy, Perceived susceptibility, and Perceived severity).
- H6: Self-efficacy is positively correlated with the perceived influence of the platform in cancer prevention.

## Methods and measures

The survey, specifically designed by the researchers for this study, was distributed through Facebook and WhatsApp, which are the two main social media platforms used by the Zurak Cancer Foundation. Independent variables were drawn from IPT and included source (credibility, trustworthiness, expertise), channel, and message characteristics. Dependent variables were drawn from HBM and included perceived susceptibility, self-efficacy, and perceived severity. Other outcome variables that measured the influence of social media platforms included the influence of the platform in action taking and perceived help in decision making for cancer prevention. Informed consent was obtained from all participants, confirming their voluntary participation and participation and understanding of the study’s purpose and procedures.

The survey instrument, which is central to our data collection and analysis, has been included as a supplementary file. Table 1 presents descriptive information of the variables employed. Internal consistency of the indices are assessed using the Cronbach’s alpha.

**Table 1:** Descriptive information

Variable	Mean (M)	Standard deviation (SD)	Cronbach’s ɑ
Social media usage	3.172	0.592	0.63
Use of social media for health info.	2.96	0.57	0.82
Message characteristics	3.872	1.77	0.74
Perceived severity	4.69	0.63	0.80
Self-efficacy	3.41	0.87	0.77
Perceived susceptibility	1.42	0.49	0.64
Message influence	3.84	0.68	0.74
Behavioral change	3.65	0.82	0.82

Table 1 above displays the summary statistics of some the indices created for the analysis. The mean, standard deviation, and Cronbach’s alpha measure of reliability are presented in columns 2,3, and 4 respectively

To gauge the extent of social media usage, respondents are asked to rate their usage of about 13 social media platforms, each on a scale of 1 (never) to 5 (Always). The social media platforms include Facebook, Facebook messenger, Instagram, Linkedin, Twitter, Whatsapp, Snapchat, WeChat, Youtube, Tiktok, Reddit, Google, and QQ. Based on the responses, about 90% of respondents stated that they have never used WeChat, Reddit, QQ, and Tiktok. As a result, these platforms are dropped from our analysis. A standerdized index of media usage ranging from 1 to 5 is generated from the remaining 9 platforms (M = 3.17, SD = 0.59, Cronbach’s ɑ = 0.63). Although this the Cronbach alpha for this index is low, it must be noted that this index is more likely a formative index rather than a reflective one. Formative indices are created from a combination of multiple factors where the individual factors are considered as causing the overall index. In our context, our index of social media usage is simply a summation of the frequency of the usage of individual social media applications, where the overall index depends on the usage of the individual apps. As Stadler et al. (2021) and Taber (2018) state, the Cronbach alpha is not a reliable measure of reliability for formative indices. Diamantopoulos & Siguaw (2006) suggest the use of the Variance Inflation Factor (VIF) to assess formative indices. A VIF value greater than 3.3 is considered problematic. The VIF for or index is 1.41, implying low multicollinearity in our construct.

The usefulness of social media as a source of health information is measured by asking respondents to rate on a scale of 1 (never) to 5 (Always) the extent to which these social media platforms provide information about health issues in general. A single scale is generated from the responses to these questions (M = 2.96, SD = 0.57, Cronbach’s ɑ = 0.82). The VIF for this construct is 1.84.

Perceived characteristics of the messages sent by the Zurak foundation is assessed by asking respondents to rate the messages from the foundation on a scale of 1 (Strongly disagree) to 5 (strongly agree) based on the following 12 characteristics; appealing, informative, useful, convincing, demonstrates seriousness of cancer in Ghana, encouraging, applicable in real life situations, and messages being repeated. A single scale is built from these responses (M = 3.87, SD = 1.77, Cronbach’s ɑ = 0.74). The VIF for this construct is 1.83.

To measure perceived severity of cancer, respondents are asked to rate on a 5-point scale, the extent to which they agree or disagree with two statements on the severity of cancer. A single scale is built from these responses. The index has a mean of 4.66 with a standard deviation of 0.63 (Cronbach’s ɑ = 0.80). Similarly, an index of self-efficacy is built from 3 questions (M = 3.41, SD = 0.87, Cronbach’s ɑ = 0.77). Similarly, an index is built to measure the perceived susceptibility to cancer from 5 questions (M = 1.42, SD = 0.49, Cronbach’s ɑ = 0.64).

Message influence is also measured by building an index from 5 questions on how the messages from the platform influenced the likelihood that they visit a physician, seek more information, speak to friends and family about cancer, share the information with others online, or skip the information. Each item is rated on a scale of 1 (Very unlikely) to 5 (Very likely). The index has a mean of 3.84 (SD = 0.68; Cronbach’s ɑ = 0.74). Behavioral change is measured by building an index using four questions that ask respondents the extent to which they agree or disagree that messages from the foundation have helped them seek early screening, reduce their fear of cancer, educate others on cancer, and clear any misconceptions about cancer. The index has a mean of 3.65 (SD = 0.821; Cronbach’s ɑ = 0.83).

The validity of the following theoretical constructs; perceived susceptibility, perceived severity, self-efficacy, message influence, and behavioral change are assessed through a Confirmatory Factor Analysis (CFA). These results for the goodness of fit tests for these constructs are provided in Table 2 The goodness of fit tests include the Chi-squared test ($\:{\chi\:}_{}^{2}$), the Comparative Fit Index (CFI), the Tucker-Lewis Index (TLI), and the Root Mean Square Error of Approximation (RMSEA). For the Chi-square test, an insignificant test statistic at 5% level of significance is a sign of good fit. Also, a CFI > 0.95 and a TLI > 0.95 implies good fit and a RMSEA < 0.05 is an indication of a strong fit. From Table 2, all the tests suggest strong fit for all the constructs.

**Table 2:** Goodness of fit tests for CFA analysis of theoretical constructs

Variable	Chi-square test	*p*-value	TLI	CFI	RMSEA	90%CL
Perceive severity (P_SERV)	0.127	0.939	1.00	1.029	0.00	0.00-0.025
Self-efficacy (SELF_EFF)	0.09	0.98	1.00	1.00	0.000	0.000–0.000
Perceived susceptibility (P_SUS)	0.174	0.418	1.00	1.00	0.000	0.088–0.723
Message influence (M_INF)	0.14	0.85	1.00	1.00	0.000	0.000–0.000
Behavioral change (B_CHN)	0.11	0.94	1.00	1.00	0.000	0.000–0.000

Table 2 above presents the demographic characteristics of the respondents. For each demographic that is categorical, the count of the number of respondents under each category as well as the corresponding percent share are reported. For demographic characteristics with continuous values, the mean, standard deviation, and rage are reported

The stated hypotheses are tested using partial correlation analysis. This method is preferred to correlation analysis because of concerns of spuriousness. Spurious correlations could occur when the relationship between two variables is driven by other variables. For example, with regard to this study, it is possible that the correlation between message characteristics and cancer prevention behavior is driven by some demographic factors such as age, education level, gender, and type of residence. When these are ignored, the correlation coefficient might show a significant association between message characteristics and cancer prevention behavior which may not be the case if these demographic factors were accounted for. By estimating partial correlations, we are able to control for demographic characteristics, while estimating the required correlations. Specifically, we control the age, educational level, gender, and the type of residence of respondents.

### Demographic characteristics

The sample contains 401 respondents (*N* = 401). About 62% of the respondents are male with the average age of a respondent being 28.6 years (Table 3). The youngest respondent is 18 years old and the oldest is 70 years old. Most of the respondents are urban dwellers (68.58%). In terms of educational background, most of the respondents have at least a bachelor’s degree (67.65%). Only about 42.14% of the respondents are familiar with the Zurak Cancer Foundation.

**Table 3:** Demographic characteristics

	Characteristics	Count (*n*)	*n* (%)
Sex	Male	247	62.1%
	Female	152	37.9%
Nationality	Ghanaian	389	97.01%
	Other countries	12	2.99%
Residence	Urban	275	68.58%
	Rural	126	31.42%
Education	Bachelor’s Degree	207	67.65%
	Postgraduate degrees	64	20.92%
	Senior High School	27	8.82%
	Junior High School	2	0.65%
	Other Certificates	6	1.96%
Familiarity with zurak foundation	Familiar	169	42.14%
	Not Familiar	232	57.86%
Age	Mean	28.61	
	Standard deviation	5.22	
	Range	52	

Table 3 above presents the partial correlation coefficients between message characteristics and social media usage, and cancer prevention behaviors. A correlation value with ** implies statistical significance at 5% level of significance, and a value with *** implies a statistical significance at 1% level of significance

On average about 82% of respondents are either moderately interested or very interested in cancer issues.

### Hypothesis testing

The stated hypotheses are tested by estimating partial correlation coefficients. As stated earlier, the correlations are computed while controlling for the age, educational level, gender, and the type of residence of respondents.

H1 predicted that social media usage is positively correlated with the source characteristics. To test this hypothesis, we estimate the partial correlation between source characteristics and social media usage. The results are presented in column 1 of Table 4. We find that social media usage is positively correlated with source credibility ($\:{r}_{partial}$=0.18**). There are no significant correlations with source attractiveness, source trustworthiness, and source expertise.

**Table 4:** Partial correlation

	Social media usage	Behavioral change	Message influence
Source credibility	0.18**	0.28***	0.34***
Source attractiveness	0.02	0.17**	0.22***
Source trustworthiness	0.10	0.07	0.06
Source expertise	0.05	0.37***	0.33***
Message characteristics index	0.01	0.37***	0.32***
Use of social media for health info.	0.23***	0.13	0.28***

**p < 0.05, ***p < 0.01

Table 4 above presents partial correlation coefficients between the HBM variables and the index of message characteristics, and the measures of cancer prevention behavior. A correlation value with ** implies statistical significance at 5% level of significance, and a value with *** implies a statistical significance at 1% level of significance

H2 predicts a positive correlation between source characteristics and cancer prevention behavior. We test this by estimating the partial correlation between the source characteristics and the message influence and behavioral change scales. Columns 2 and 3 of Table 4 contain these results. We find positive and significant correlations between the behavioral change scale and source credibility ($\:{r}_{partial}$=0.28***), attractiveness($\:{r}_{partial}$=0.17***), and expertise ($\:{r}_{partial}$=0.37***). There are no significant correlations between behavioral change and source trustworthiness. We also find positive and significant correlations between message influence index and source credibility($\:{r}_{partial}$=0.34***), attractiveness($\:{r}_{partial}$=0.22***), and expertise($\:{r}_{partial}$=0.33***). These imply that when people perceive the platforms as credible, attractive, or having the right expertise, they are more likely to take the messages more seriously and are more likely to change their behavior toward cancer prevention.

H3 predicted that the perception of the message is correlated with the influence of cancer prevention behavior. To test this hypothesis, we estimate the partial correlation between the index of message characteristics and cancer prevention behavior. From Table 4, we find a positive and significant correlation between the index of message characteristics and the behavioral change index ($\:{r}_{partial}$=0.366***). Similarly, a significant positive correlation exist between the message characteristics index and the message influence index ($\:{r}_{partial}$=0.32***)

We proceed to test H4 by estimating the partial correlation between perceptions of social media as a source of health information and message influence and behavioral change indices. These are also presented in Table 4. We find positive and significant correlations between the index of social media as a source of health information and the message influence index ($\:{r}_{partial}$=0.28**). This implies that those who rely on social media as a source of health information are more likely to be associated with a change cancer-prevention behavior.

To test H5, we test the correlation between the message characteristics index and the variables in the HBM model namely, self-efficacy, perceived susceptibility, and perceived severity. These partial correlations are presented in column 1 of Table 5. There are no significant correlations between the variables in the HBM model and the message characteristics index.

**Table 5:** Partial correlation between HBM variables and Cancer prevention behavior

	Message characteristics index	Behavioral change	Message influence	Social media usage
Self-efficacy	0.10	0.40***	0.29***	0.13**
Perceived susceptibility	−0.09	0.03	−0.18**	0.02
Perceived severity	0.11	0.02	0.10	0.17***

**p < 0.05, ***p < 0.01

Finally, we test H6 to identify if self-efficacy is positively correlated with the perceived influence of the platform in cancer prevention. They are provided in columns 2 and 3 of Table 5. We find significant correlations between self-efficacy and the behavioral change index ($\:{r}_{partial}$=0.40**) and the message influence index ($\:{r}_{partial}$=0.29***).

We also test for the correlation between social media usage and self-efficacy, preserved susceptibility, and perceived severity. Our results also prided in Table 5 show that social media usage is positively correlated with self-efficacy ($\:{r}_{partial}$=0.13**) and perceived severity ($\:{r}_{partial}$=0.17***). We do not find any significant correlation between social media usage and perceived susceptibility to cancer.

### Multiple regression results

For purposes of robustness, we also conduct a multiple regression estimations to confirm the partial correlations used to test out hypothesis. For each hypothesis, we estimate a multiple regression to test the hypothesis. Across all estimations, we control for the same demographic characteristics that were controlled for in the partial correlations. From Table 6, we can observe that not association between social media usage and source attractiveness, source expertise and source trustworthiness. However, there is a strong association between social media usage and source credibility. This is similar to the results for the partial correlations.

**Table 6:** Regression estimates of the relationship between source characteristics and social media usage

	(1)	(2)	(3)	(4)
VARIABLES	Source attractiveness	Source credibility	Source expertise	Source trustworthiness
smedia	0.0308	0.278***	0.0802	0.223
	(0.199)	(0.0959)	(0.0886)	(0.129)
age	−0.0865***	0.0225	−0.00449	0.0244
	(0.0302)	(0.0211)	(0.0164)	(0.0250)
urban	0.387	0.340*	0.406*	−0.0796
	(0.411)	(0.203)	(0.214)	(0.217)
Senior high school graduate	0.836	−0.557**	−0.776***	−0.472
	(1.013)	(0.281)	(0.276)	(0.297)
Bachelor’s degree or diploma	0.661	−0.688***	−0.860***	−0.759***
	(0.942)	(0.218)	(0.196)	(0.173)
Postgraduate	0.685	−0.752***	−1.109***	−0.793***
	(0.978)	(0.258)	(0.228)	(0.231)
female	−0.566**	0.112	0.0402	0.0242
	(0.268)	(0.138)	(0.110)	(0.159)
Constant	1.745	3.146***	4.642***	3.532***
	(1.457)	(0.795)	(0.635)	(1.063)
Observations	299	162	162	160
R-squared		0.074	0.078	0.034

Robust standard errors in parentheses

*** *p* < 0.01, ** *p* < 0.05, * *p* < 0.1

In Tables 7 and 8, hypothesis 2 is tested by multiple regressions. Just like in the partial correlation analysis, we find statistically significant associations between the source characteristics; source credibility, source expertise and source attractiveness, and the behavioral change and message influence indices. There is no significant association between source trustworthiness and the behavioral change and message influence indices.

**Table 7:** Regression estimates of the relationship between source characteristics and behavioral change

	(1)	(2)	(3)	(4)
VARIABLES	Behavioral change	Behavioral change	Behavioral change	Behavioral change
Source credibility	0.288***			
	(0.0730)			
Source attractiveness		0.823***		
		(0.278)		
Source trustworthiness			0.0647	
			(0.0732)	
Source expertise				0.467***
				(0.0985)
age	−0.0129	−0.00718	−0.0112	−0.00557
	(0.0179)	(0.0189)	(0.0189)	(0.0171)
urban	−0.288*	−0.214	−0.215	−0.375**
	(0.167)	(0.152)	(0.171)	(0.177)
Senior high school graduate	−0.620	−0.796*	−1.286***	−0.433
	(0.458)	(0.406)	(0.249)	(0.493)
Bachelor’s degree or diploma	−0.696	−0.864**	−1.371***	−0.505
	(0.429)	(0.370)	(0.190)	(0.452)
Postgraduate	−0.885*	−1.107***	−1.583***	−0.598
	(0.453)	(0.392)	(0.199)	(0.471)
female	−0.0444	0.0129	−0.0369	−0.0378
	(0.138)	(0.142)	(0.143)	(0.131)
Constant	3.744***	4.122***	5.286***	2.667***
	(0.854)	(0.791)	(0.818)	(0.873)
Observations	162	162	160	162
R-squared	0.116	0.071	0.050	0.171

Robust standard errors in parentheses

*** *p* < 0.01, ** *p* < 0.05, * *p* < 0.1

**Table 8:** Regression estimates of the relationship between source characteristics and message influence

	(1)	(2)	(3)	(4)
VARIABLES	Message influence	Message influence	Message influence	Message influence
Source credibility	0.288***			
	(0.0707)			
Source attractiveness		0.878***		
		(0.295)		
Source trustworthiness			0.0431	
			(0.0701)	
Source expertise				0.347***
				(0.0823)
age	−0.00654	−0.000682	−0.00557	1.03e-05
	(0.0149)	(0.0168)	(0.0166)	(0.0158)
urban	−0.164	−0.0931	−0.126	−0.202
	(0.178)	(0.179)	(0.183)	(0.190)
Senior high school graduate	−0.0187	−0.193	−1.163***	0.0715
	(0.752)	(0.704)	(0.212)	(0.760)
Bachelor’s degree or diploma	0.0399	−0.125	−1.115***	0.125
	(0.741)	(0.687)	(0.144)	(0.746)
Postgraduate	−0.216	−0.436	−1.398***	−0.0650
	(0.755)	(0.698)	(0.183)	(0.758)
female	−0.0316	0.0281	−0.0360	−0.0218
	(0.114)	(0.116)	(0.116)	(0.114)
Constant	2.959***	3.275***	5.125***	2.478**
	(0.963)	(0.930)	(0.685)	(0.999)
Observations	162	162	160	162
R-squared	0.138	0.078	0.056	0.135

Robust standard errors in parentheses

*** *p* < 0.01, ** *p* < 0.05, * *p* < 0.1

In Table 9, hypothesis 3 is tested and the results are in contrast to the partial correlation results. While the partial correlation results showed significant positive correlation between the index of message characteristics and the measures of cancer prevention behavior, the multiple regression results show no significant associations between message characteristics and cancer prevention behavior.

**Table 9:** Relationship between perception of the message and cancer prevention behavior

	(1)	(2)
VARIABLES	Message influence	Behavioral change
Message characteristics	0.0172	0.00949
	(0.0322)	(0.0364)
age	−0.00258	−0.00886
	(0.0170)	(0.0191)
urban	−0.0530	−0.179
	(0.188)	(0.173)
Senior high school graduate	−0.226	−0.819*
	(0.715)	(0.415)
Bachelor’s degree or diploma	−0.206	−0.931**
	(0.698)	(0.385)
Postgraduate	−0.489	−1.145***
	(0.711)	(0.409)
female	−0.0219	−0.0303
	(0.117)	(0.141)
Constant	4.171***	4.975***
	(0.871)	(0.710)
Observations	162	162
R-squared	0.031	0.043

Robust standard errors in parentheses

*** *p* < 0.01, ** *p* < 0.05, * *p* < 0.1

Hypothesis 4is tested in Table 10. Here, we find significant positive correlation between the index of social media as a source of health information and the behavioral change index while association with the message influence index is positive but insignificant. Even though the partial correlation results showed positive correlations as well, the association with the message influence index was significant while that of the behavioral change index was not significant.

**Table 10:** Relationship between perceptions of social media as a source of health information and cancer prevention behavior

	(1)	(2)
VARIABLES	Message influence	Behavioral change
Use of social media for health info.	0.158	0.415***
	(0.107)	(0.139)
age	0.000549	−0.00117
	(0.0162)	(0.0170)
urban	−0.0606	−0.187
	(0.189)	(0.167)
Senior high school graduate	−0.11	−0.561
	(0.745)	(0.496)
Bachelor’s degree or diploma	−0.115	−0.742
	(0.727)	(0.464)
Postgraduate	−0.378	−0.916*
	(0.737)	(0.484)
female	−0.0330	−0.0794
	(0.117)	(0.134)
Constant	3.589***	3.373***
	(0.966)	(0.873)
Observations	162	162
R-squared	0.045	0.115

Robust standard errors in parentheses

*** *p* < 0.01, ** *p* < 0.05, * *p* < 0.1

Hypothesis 5 is also tested by multiple regression and the results are presented in Table 11. Similar to the partial correlation results, no significant associations are detected between the variables in the HBM model and the message characteristics index.

**Table 11:** Relationship between message characteristics index and self-efficacy, perceived susceptibility, and perceived severity

	(1)	(2)	(3)
VARIABLES	Self-efficacy	Perceived susceptibility	Perceived severity
Message characteristics	0.0187	0.0179	−0.0287
	(0.0380)	(0.0183)	(0.0214)
age	0.0141	0.0130	−0.00252
	(0.0222)	(0.0107)	(0.0128)
urban	−0.136	−0.0388	0.162
	(0.205)	(0.118)	(0.108)
Senior high school graduate	0.524	0.310	−0.344**
	(0.444)	(0.195)	(0.173)
Bachelor’s degree or diploma	0.395	0.187	−0.297**
	(0.408)	(0.175)	(0.137)
Postgraduate	0.190	0.0476	−0.387*
	(0.434)	(0.191)	(0.207)
female	0.0565	−0.0206	0.125
	(0.151)	(0.0696)	(0.105)
Constant	2.728***	0.785**	5.009***
	(0.808)	(0.376)	(0.431)
Observations	162	162	162
R-squared	0.015	0.032	0.042

Robust standard errors in parentheses

*** *p* < 0.01, ** *p* < 0.05, * *p* < 0.1

Lastly hypothesis 6 is also tested and the results are presented in Table 12. Similar to the partial correlation results, the regression results show positive and significant associations between self-efficacy and the perceived influence of the platform in cancer prevention.

**Table 12:** Relationship between self-efficacy and cancer prevention behavior

	(1)	(2)
VARIABLES	Behavioral change	Message influence
Self-efficacy	0.362***	0.210***
	(0.0730)	(0.0620)
urban	−0.136	−0.0396
	(0.133)	(0.168)
Senior high school graduate	−0.920***	−0.288
	(0.231)	(0.612)
Bachelor’s degree or diploma	−1.031***	−0.260
	(0.208)	(0.595)
Postgraduate	−1.201***	−0.497
	(0.256)	(0.610)
female	−0.00580	0.00249
	(0.122)	(0.104)
Constant	3.550***	3.454***
	(0.316)	(0.632)
Observations	164	164
R-squared	0.194	0.104

Robust standard errors in parentheses

*** *p* < 0.01, ** *p* < 0.05, * *p* < 0.1

## Discussion

This study aimed to assess persuasion within the Zurak Cancer Foundation (ZCF) platform, shedding light on its influence on behavior change in Ghana. We investigate how both the source and message characteristics within ZCF shape behavior and message impact. Additionally, we explore the roles of perceived susceptibility, self-efficacy, and perceived severity in message perception and platform efficacy. The results of our analysis support H1 only for source credibility, while no significant correlations were found for source attractiveness, source trustworthiness, and source expertise. This implies that individuals tend to rely on sources they perceive as more credible when engaging with social media platforms. This finding aligns with previous studies that emphasize the importance of source credibility in shaping information consumption behaviors in online environments [77, 78]. However, our results showed that social media usage does not correlate with source attractiveness, reliability, or knowledge, raising intriguing considerations. It shows that social media users may choose source credibility over other qualities. Source attractiveness, trustworthiness, and competence may be less relevant in social media information consumption than offline.

Results showed that source characteristics are positively correlated with cancer prevention behavior and therefore H2 was supported. This indicates that individuals are more likely to engage in cancer preventive behaviors when they perceive the sources as credible, attractive, and knowledgeable in the domain of cancer prevention. The findings indicate that social media platforms, including ZCF, possess the capacity to exert influence over users provided they are perceived as credible. The credibility, attractiveness, and expertise of the sources enhance the persuasive impact of the messages, leading individuals to perceive them as more influential in shaping their behavior [79]. Although some studies have not demonstrated the influence of source credibility, several studies have suggested that perceiving social media as credible may facilitate the adoption of preventive behaviors [77, 78]. This suggests that, in the context of digital health communication, users may prioritize credibility over other traditional source cues such as attractiveness or trustworthiness. Such findings point to potential differences between online and offline source evaluations, where visual cues or interpersonal familiarity may play a more dominant role.

Our results further supported H3 thus demonstrating positive and statistically significant partial correlations between specific message characteristics and behavioral change. This means that messages that are appealing, informative, useful, convincing, encouraging, and address multiple cancer types while being applicable to real-world situations are associated with a higher likelihood of behavior change. This aligns with previous studies that have also identified a positive relationship between message characteristics and behavioral change [80, 81]. These findings are consistent with McGuire’s theoretical framework, which suggests that various message features, including the use of humor, fear, or control, as well as the frequency of message repetition, play a crucial role in enhancing message efficacy and facilitating behavior change [56, 66]. However, it is important to note that this association was observed only in the partial correlation analysis; the multiple regression results did not replicate this finding. This discrepancy suggests that when other factors are controlled for simultaneously, the unique contribution of message characteristics to behavior change may be diminished. This calls for caution in interpreting bivariate associations and highlights the complexity of understanding the pathways through which messages influence health behaviors.

Our research results revealed a significant correlation between social media as a source of health information and both the message influence index and the behavioral change index which supports our fourth hypothesis. This indicates that individuals who rely on social media platforms for health information perceive the messages they encounter as influential and are more likely to engage in cancer prevention behavior change. The impact of social media on individuals’ attitudes, beliefs, and intentions regarding cancer prevention behavior is noteworthy. This result is consistent with studies that found that the accessibility, wide reach, and interactive nature of social media platforms contribute to their influence in shaping individuals’ health-related perceptions and cancer prevention behaviors [82, 83]. However, multiple regression analysis revealed that only the association with behavioral change remained significant, while the association with message influence became non-significant. This suggests that although individuals may act on the information they receive from social media, they may not necessarily perceive those messages as highly influential. This finding implies that actual behavior change may be driven by factors beyond message impact perception, such as habitual platform use or perceived personal relevance.

The present study provides further evidence supporting a positive and statistically significant relationship between variables of the Health Belief Model (HBM)– namely self-efficacy, perceived susceptibility, and perceived severity– and specific message characteristics, including informativeness, usefulness, convincingness, demonstration of cancer seriousness in Ghana, encouragement, addressing different types of cancer, and applicability to real-life situations. In terms of perceived susceptibility, the findings indicate a positive and significant correlation with the perception that messages are lengthy, suggesting that individuals who perceive messages as lengthy are more likely to perceive themselves as susceptible to cancer [71]. The lengthiness of the messages may convey a sense of comprehensive information coverage, thereby increasing individuals’ perception of susceptibility. However, it is important to note that the lack of significant correlations between perceived susceptibility and other message characteristics suggests that additional factors beyond those examined in this study may influence individuals’ perceived susceptibility to cancer.

These findings suggest that message attributes and framing have the potential to influence individuals’ perceptions of cancer susceptibility, severity, and self-efficacy. This aligns with previous research conducted by [84], who found a significant relationship between messages and self-efficacy for smoking cessation, with sustained increases observed in self-efficacy among individuals exposed to loss-framed messages. Similarly, a study by [85] demonstrated that individuals with high self-efficacy were more likely to engage in skin self-examination when exposed to loss-framed messages compared to gain-framed messages.

Finally, our sixth hypothesis stated that self-efficacy would be positively correlated with the perceived influence of the platform. However, the results only reveal a significant correlation between self-efficacy and the behavioral change index. No significant correlation was found between self-efficacy and the message influence index [86].

The observed correlation between self-efficacy and the behavioral change index suggests that individuals exhibiting greater self-efficacy are more inclined to undertake behavioral modifications aimed at preventing cancer. Self-efficacy pertains to an individual’s conviction in their capability to effectively execute a behavior [86].The present discovery implies that individuals possessing a greater sense of self-efficacy exhibit greater assurance in their capacity to initiate and maintain behavioral modifications.

Nevertheless, it is noteworthy that a substantial correlation between self-efficacy and the message influence index was not observed. This may imply that although self-efficacy contributes to the advancement of factual behavioral modification, it may not be inherently linked to the perceived impact of the platform or the effectiveness of the communicated messages. It is plausible that additional variables beyond self-efficacy, such as the credibility or appeal of the platform, may exert a greater impact on individuals’ evaluations of message efficacy.

The results enhance comprehension of the correlation among self-efficacy, perceived platform influence, and behavioral modification within the realm of cancer prevention. Self-efficacy plays a crucial role in motivating tangible modifications in behavior, yet its impact may operate autonomously from individuals’ assessments of message efficacy. The statement suggests that cancer prevention interventions and campaigns should not exclusively prioritize the enhancement of self-efficacy. Rather, they should consider other factors that influence individuals’ perceptions of message efficacy and persuasive communication.

### Theoretical, methodological, and practical implications (All)

These findings have implications for both researchers and practitioners. Researchers can further explore the reasons behind the observed positive correlation between social media usage and source credibility, as well as investigate other potential factors that may influence individuals’ information consumption behaviors on social media. Practitioners, such as media organizations and content creators, can leverage the importance of source credibility to enhance the impact of their social media presence. By establishing and maintaining a reputation for credibility, they can attract and engage a larger audience in an era where social media plays a pivotal role in information dissemination.

The findings have important implications for health communication and intervention strategies. Health campaigns and initiatives aimed at promoting cancer preventive behaviors should prioritize the selection of sources that are perceived as credible, attractive, and knowledgeable in the domain of cancer prevention. By leveraging the power of these source characteristics, health messages can be tailored to enhance their pervasiveness and effectiveness in motivating behavior change.

In addition, this study reveals noteworthy associations between social media utilization for health-related purposes and two key indices, namely the message influence index and the behavioral change index. These findings underscore the substantial influence of social media platforms in shaping individuals’ attitudes, beliefs, and intentions towards cancer prevention behavior. Furthermore, the observed positive associations between distinct message attributes and alterations in behavior underscore the importance of proficient message construction in advancing favorable health consequences. Finally, the main theoretical innovation of this study lies in the integration of the Information Processing Theory (IPT) and the Health Belief Model (HBM), offering a comprehensive framework that bridges message design with individual health beliefs to explain cancer prevention behavior. While IPT highlights how source credibility, message characteristics, and media channels shape message reception and engagement, HBM focuses on internal cognitive factors such as perceived susceptibility, severity, and self-efficacy that motivate health actions. By combining these models, the study captures both the external and internal drivers of behavior change, providing deeper insights into how social media messages influence cancer prevention in Ghana. This dual-framework approach is especially useful for a study of this nature, as it allows for a more holistic assessment of how digital platforms like the Zurak Cancer Foundation’s social media content impact not just what people see and trust, but how they think, feel, and ultimately act. It enhances the ability to design culturally relevant, psychologically informed health interventions tailored to digitally connected, underserved populations. The findings of this research exhibit a high degree of conformity with both the Information Processing Theory and the Health Belief Model. These theoretical frameworks are valuable in comprehending the fundamental mechanisms that underpin the impact of social media on health-related behaviors.

### Limitations (All)

Although the aforementioned research offers significant perspectives on the associations among diverse factors, it is crucial to acknowledge its constraints. The research utilizes a cross-sectional design, which entails the collection of data at a particular moment in time. The presence of this particular constraint hinders the ability to establish causal relationships among variables. To establish cause-and-effect relationships, longitudinal or experimental designs would be more suitable.

The present study is dependent on self-report measures, which are susceptible to memory recall difficulties and social desirability bias. Respondents may exhibit a tendency to provide answers that align with social norms or may encounter challenges in accurately recollecting their behaviors and perceptions. One potential limitation of the study is the absence of control for confounding variables. While the study does control for demographic characteristics, there may be additional factors that could impact the relationships between variables that were not considered. The presence of omitted variables has the potential to introduce confounding factors into the observed associations.

Importantly, a key limitation of this study is that we did not collect data on the specific types of cancer. This limits the scope of our findings and may affect their generalizability. Future research should consider exploring multiple cancer types to provide a more comprehensive understanding of cancer-related experiences and perceptions.

The investigation centers on a restricted range of source characteristics, including but not limited to credibility, attractiveness, trustworthiness, and expertise. Additional source attributes that may impact social media utilization and modification of behavior may not have been taken into account. The research did not account for extraneous variables or contextual factors that could potentially impact social media utilization and modifications in behavior. The observed relationships may be confounded by various factors, including but not limited to cultural norms, social support, and exposure to other health promotion campaigns.

The research is centered on a particular domain, namely the prevention of cancer, and a specific demographic group, namely individuals from Ghana. The generalizability of the results to different health settings or heterogeneous demographics may be limited.

It is imperative to take into account the constraints outlined in the study’s results interpretation and to promote additional research endeavors aimed at tackling these limitations.

## Supplementary Information

Supplementary Material 1.

## Data Availability

The datasets generated and/or analyzed will be made available from the corresponding author upon request.
