# Supplementary material for: Enhancing cancer prevention behaviors through social media: the role of source credibility and message characteristics in Ghana
Source: BMC Public Health. 2025 Jul 16;25:2471. doi: 10.1186/s12889-025-23693-1 (PMC12265361; doi:10.1186/s12889-025-23693-1)
Supplement: Supplementary file 1 — Supplementary Material 1. [file 12889_2025_23693_MOESM1_ESM.docx]

Cancer project

Start of Block: Default Question Block

The purpose of this survey is to gather your opinions on cancer health communications in Ghana. This is an academic research study conducted by a graduate student in the A.Q.Miller School of Journalism and Mass Communications at Kansas State University. Your anonymous responses will be kept strictly confidential. You will only be asked for your opinions and your honest responses are very important for this study so please respond to all questions as honestly as possible. The study does not have any foreseeable risk to you but if you feel some kind of discomfort you may discontinue your participation from the study at any time. This project is approved by the Institutional Review Board (IRB # 10167) at Kansas State University.
 
 TERMS OF PARTICIPATION: I understand this project is research, and that my participation is completely voluntary. I also understand that if I decide to participate in this study, I may withdraw my consent at any time, and stop participating at any time without explanation, penalty, or loss of benefits, or academic standing to which I may otherwise be entitled.
 
I verify by clicking “I Accept” below, I indicate that I have read and understand this consent form, and willingly agree to participate in this study under the terms described.

- I Accept (1)
- Do not accept (2)

Skip To: End of Survey If The purpose of this survey is to gather your opinions on cancer health communications in Ghana. T... = Do not accept

Q1 **Are you a Ghanaian?**

- Yes (1)
- No (2)

Skip To: End of Survey If Are you a Ghanaian? = No

Q2 **Do you currently live in Ghana?**

- Yes (1)
- No (2)

Q3 **Who were you with when you began completing this survey?**

- Alone (1)
- Friends (2)
- Family (3)
- Colleagues (4)
- Strangers (5)
- Other (6)

Q4 **Where do you find information on cancer health? - select all that apply**

- Social media (e.g. Facebook, Twitter, WhatsApp etc.) (1)
- Traditional media (Radio, Newspapers, Television, Magazines (2)
- Information leaflets, books, magazines (3)
- Family, friends, or other acquaintances (4)
- Medical or health professionals (e.g. doctor, nurse) (5)
- Others (6) __________________________________________________

| Page Break |  |
| --- | --- |

Q5 **Generally speaking, how interested are you in cancer health issues?**

- Not at all interested (1)
- Slightly interested (2)
- Moderately interested (3)
- Very interested (4)
- Extremely interested (5)

| Page Break |  |
| --- | --- |

Q6 **How concerned are you about cancer**

- Not at all concerned (1)
- Slightly concerned (2)
- Moderately concerned (3)
- Very concerned (4)
- Extremely Concerned (5)

| Page Break |  |
| --- | --- |

Q7 **Please indicate the extent to which you agree with the following statements.**
 If you are using a phone, please click on this scroll symbol to see all the choices.

|  | Strongly Disagree (1) | Disagree (2) | Neither agree nor disagree (3) | Agree (4) | Strongly agree (5) |
| --- | --- | --- | --- | --- | --- |
| I have a history of cancer in my family. (1) |  |  |  |  |  |
| I have been diagnosed with a serious illness that weakens my immune system. (2) |  |  |  |  |  |
| I have never been screened for any type of cancer and don’t know my status. (3) |  |  |  |  |  |
| I have been diagnosed with a severe illness that I thought was cancer. (4) |  |  |  |  |  |
| I think cancer is a spiritual problem. (5) |  |  |  |  |  |
| I use alcohol and tobacco products (6) |  |  |  |  |  |
| I got diagnosed with cancer (7) |  |  |  |  |  |

| Page Break |  |
| --- | --- |

Q8
**How strongly do you agree or disagree with the following statements.**

|  | Strongly Disagree (1) | Disagree (2) | Neither agree nor disagree (3) | Agree (4) | Strongly agree (5) |
| --- | --- | --- | --- | --- | --- |
| Cancer is a very serious health problem. (1) |  |  |  |  |  |
| Cancer can have very serious effects on ones health. (2) |  |  |  |  |  |
| Cancer is very widespread in Ghana. (3) |  |  |  |  |  |
| Cancer has high rate of death in Ghana. (4) |  |  |  |  |  |
| Cancer cannot be treated in Ghana. (5) |  |  |  |  |  |
| Cancer only affects the elderly. (6) |  |  |  |  |  |
| Cancer is very expensive to trreat in Ghana (7) |  |  |  |  |  |

| Page Break |  |
| --- | --- |

Q9
**Please indicate the extent to which you agree or disagree with the following statements:**
 

|  | Strongly Disagree (1) | Disagree (2) | Neither agree nor disagree (3) | Agree (4) | Strongly agree (5) |
| --- | --- | --- | --- | --- | --- |
| I can get screened for different types of cancer if I want to. (1) |  |  |  |  |  |
| I know where to go and get tested if I want. (2) |  |  |  |  |  |
| I am confident about where to go for testing and treatment. (3) |  |  |  |  |  |
| I can afford cancer treatment if i need it. (4) |  |  |  |  |  |
| I am confident that cancer can be cured if detected early. (5) |  |  |  |  |  |

| Page Break |  |
| --- | --- |

Q10 **Name 3 types of cancer that you believe are the most serious:**

________________________________________________________________

________________________________________________________________

________________________________________________________________

________________________________________________________________

________________________________________________________________

Q12 **Rate your use of these social media platforms.**

|  | Never (1) | Rarely(few times a month (2) | Sometimes (Few times a week) (3) | Often ( several times a day) (4) | Always (several times an hour) (5) |
| --- | --- | --- | --- | --- | --- |
| Facebook (1) |  |  |  |  |  |
| Facebook Messenger (2) |  |  |  |  |  |
| Instagram (3) |  |  |  |  |  |
| LinkedIn (4) |  |  |  |  |  |
| Twitter (5) |  |  |  |  |  |
| Whatsapp (6) |  |  |  |  |  |
| Snapchat (7) |  |  |  |  |  |
| WeChat (8) |  |  |  |  |  |
| YouTube (9) |  |  |  |  |  |
| Tiktok (10) |  |  |  |  |  |
| Reddit (11) |  |  |  |  |  |
| Google (12) |  |  |  |  |  |
| QQ (13) |  |  |  |  |  |

Q13 **Please indicate the extent to which these social media platforms provide information about health issues in general (not necessarily information about cancer).**

|  | Strongly Disagree (1) | Disagree (2) | Neither agree nor disagree (3) | Agree (4) | Strongly agree (5) |
| --- | --- | --- | --- | --- | --- |
| Facebook (1) |  |  |  |  |  |
| Facebook Messenger (2) |  |  |  |  |  |
| Instagram (3) |  |  |  |  |  |
| LinkedIn (4) |  |  |  |  |  |
| Twitter (5) |  |  |  |  |  |
| WhatsApp (6) |  |  |  |  |  |
| SnapChat (7) |  |  |  |  |  |
| WeChat (8) |  |  |  |  |  |
| YouTube (9) |  |  |  |  |  |
| Tiktok (10) |  |  |  |  |  |
| Reddit (11) |  |  |  |  |  |
| Google (12) |  |  |  |  |  |
| QQ (13) |  |  |  |  |  |

| Page Break |  |
| --- | --- |

Q14 **Are you familiar with Zurak cancer Foundation?**

- Yes (1)
- No (2)

Skip To: Q22 If Are you familiar with Zurak cancer Foundation?   = No

Q15 **How credible do you find their platform?  Please rate it on a scale of 1 (not at all credible) to 5 (very credible)**

|  | 1 | 2 | 3 | 4 | 5 |
| --- | --- | --- | --- | --- | --- |

| Not credible () | 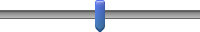 |
| --- | --- |

Q16 **Do you find this platform to be attractive?**

- Yes (1)
- No (2)

Q17 **How Trustworthy do you find this platform?  (Please rate it on a scale of 1 (not at all trustworthy to 5 (very trustworthy)**

|  | 1 | 2 | 3 | 4 | 5 |
| --- | --- | --- | --- | --- | --- |

| Not trustworthy () | 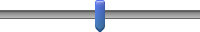 |
| --- | --- |

Q18 **Zurak Cancer Foundation has the expertise needed to provide valuable information about cancer**

- Strongly Disagree (1)
- Disagree (2)
- Neither agree nor disagree (3)
- Agree (4)
- Strongly agree (5)

Q19
 **How strongly do you agree or disagree with the following statement.**    **Messages posted by Zurak organization on this platform are:**

|  | Strongly Agree (1) | Agree (2) | Neither agree nor disagree (3) | Strongly Disagree (4) | Disagree (5) |
| --- | --- | --- | --- | --- | --- |
| Appealing (1) |  |  |  |  |  |
| Fearful (2) |  |  |  |  |  |
| Lengthy (3) |  |  |  |  |  |
| Not lengthy (4) |  |  |  |  |  |
| Informative (5) |  |  |  |  |  |
| Useful (6) |  |  |  |  |  |
| Convincing (7) |  |  |  |  |  |
| Demonstrate the seriousness of cancer in Ghana (8) |  |  |  |  |  |
| Encouraging (9) |  |  |  |  |  |
| Address different types of cancer (10) |  |  |  |  |  |
| Applicable to real life situations (11) |  |  |  |  |  |
| Repeated (12) |  |  |  |  |  |

Q20 **How likely or unlikely has cancer information on this platform influenced you?**
 **Cancer information on this platform has influenced me to:**

|  | Very Unlikely (1) | Unlikely (2) | Neutral (3) | Likely (4) | Very Likely (5) |
| --- | --- | --- | --- | --- | --- |
| visit a physician (1) |  |  |  |  |  |
| seek more information (2) |  |  |  |  |  |
| speak to my friends/family about cancer (3) |  |  |  |  |  |
| share the information with others online (4) |  |  |  |  |  |
| skip the information (5) |  |  |  |  |  |

Q21 **Thinking of how this platform has influenced you, how much do you agree or disagree with the following?**

|  | Strongly Disagree (1) | Disagree (2) | Neither agree nor disagree (3) | Agree (4) | Strongly agree (5) |
| --- | --- | --- | --- | --- | --- |
| It has helped me to seek early screening. (1) |  |  |  |  |  |
| It has helped me to reduce my fear of cancer. (2) |  |  |  |  |  |
| I rely on it to educate others on cancer. (3) |  |  |  |  |  |
| It has helped to clear my misconceptions about cancer. (4) |  |  |  |  |  |

| Page Break |  |
| --- | --- |

Q22 **What's your age in years?  (indicate the actual year of birth e.g. 1976)**

________________________________________________________________

Q23 **What is your sex?**

- Male (1)
- Female (2)

Q24 **What is the highest level of education you have completed?**

- Less than junior high school (1)
- Junior high school graduate (2)
- Senior high school graduate (3)
- Bachelor’s degree or diploma (4)
- Post graduate (5)
- Other (6)

Q25 **What kind of community do you currently live in?**

- Urban (1)
- Rural (2)

End of Block: Default Question Block
